# Early impact of the Australian national shingles vaccination program with the herpes zoster live attenuated vaccine

**DOI:** 10.1080/21645515.2020.1754702

**Published:** 2020-05-18

**Authors:** John Litt, Robert Booy, Debra Bourke, Dominic E. Dwyer, Alan Leeb, Philip McCloud, Alicia N. Stein, Michael Woodward, Anthony L. Cunningham

**Affiliations:** a Discipline of General Practice College of Medicine and Public Health, Flinders University, Adelaide, Australia; b National Centre for Immunisation Research and Surveillance of Vaccine Preventable Diseases, The University of Sydney, Sydney, Australia; c Medical Department, Seqirus (Australia) Pty Ltd, Parkville, Australia; d NSW Health Pathology – Institute for Clinical Pathology and Medical Research, Westmead Hospital and the University of Sydney, Sydney, Australia; e Illawarra Medical Centre, Ballajura, Australia; f McCloud Consulting Group, Belrose, Australia; g Helath Economics, Seqirus (Australia) Pty Ltd, Parkville, Australia; h Centre for Virus Research, Aged Care Services, Austin Health, University of Melbourne, Melbourne, Australia; i Faculty of Medicine and Health, The Westmead Institute for Medical Research, Westmead, Australia; j The University of Sydney, Sydney, Australia

**Keywords:** Herpes zoster, herpes zoster vaccine, postherpetic neuralgia, shingles, varicella zoster virus, zoster virus vaccine live

## Abstract

Herpes zoster (shingles) is a painful condition resulting from reactivation of latent varicella zoster virus (VZV). The Australian National Shingles Vaccination Program (commenced November 2016) provides free herpes zoster vaccination for eligible adults aged 70 years, with a 5-year catch-up program (until October 2021) for adults aged 71–79 years. Patterns and impact of the program were evaluated by analysis of vaccine distribution and delivery data and specific antiviral prescription data from the Pharmaceutical Benefits Scheme. During the first 2 years, uptake of funded live attenuated shingles vaccine ZOSTAVAX® (Zoster Virus Vaccine Live; ZVL) was high across the ongoing and catch-up programs. Before program implementation (2006–2016), herpes zoster coded antiviral prescription rates increased by 2.2% per year (95% CI: 1.5, 2.9) in the 70–79 years age group. In the two years since program launch, herpes zoster antiviral prescription rates declined substantially in this age group, by an average of 13.6% per year (95% CI: 1.5, 24.2). These results indicate that the National Shingles Vaccination Program has been highly successful in vaccinating a considerable proportion of Australian adults aged 70–79 years against herpes zoster and suggest that vaccine uptake was associated with decreased incidence of herpes zoster.

## Introduction

Herpes zoster (shingles) is a painful condition usually characterized by an erythematous papulovesicular rash in a unilateral dermatomal distribution in immunocompetent individuals.^1,2^ Herpes zoster results from reactivation of latent neuronal varicella zoster virus (VZV).^1,2^ The incidence of herpes zoster increases with age owing to a progressive decline in virus-specific cell-mediated immunity, leading to VZV reactivation.^1,2^ In Australia, according to data from the BEACH (Bettering the Evaluation And Care of Health) database, there were an estimated 5.6 cases per 1,000 population per year (all ages) over the period 2006–2013, with an estimated 13.7, 15.3, and 19.9 cases per 1,000 population in the 60–69 years, 70–79 years, and ≥80 years age groups, respectively.^3^ Older individuals are also more likely to develop complications from herpes zoster, including the debilitating condition of postherpetic neuralgia (PHN; defined as pain lasting more than 3 months after the onset of rash).^1,2^ One in five patients aged over 50 years with herpes zoster will continue to report pain 6 months after the onset of rash despite adequate antiviral therapy.^4^ Although less common, increased risk of stroke and other cardiovascular events is also reported following an episode of herpes zoster.^5-8^ The economic burden of herpes zoster and PHN is high in Australia, with estimated annual costs to the healthcare system of approximately AUD 33 million in 2006 for people aged 50 years and older.^9^

The herpes zoster vaccine ZOSTAVAX® (ZVL) is a live attenuated vaccine containing the Oka/Merck strain of VZV, the same strain used in the childhood VZV vaccine against chickenpox, but the amount of virus antigen is approximately 14 times greater.^1,10^ The Shingles Prevention Study, a randomized, double-blind, placebo-controlled study conducted in 38,546 adults aged ≥60 years, showed that ZVL significantly reduced the incidence of both herpes zoster and PHN and also reduced the severity of herpes zoster in those who did develop the disease.^10^ Over a median follow-up of 3.1 years, vaccine efficacy (VE) against herpes zoster and PHN was 51% and 67%, respectively, and the burden of illness associated with herpes zoster was reduced by 61%.^10^ VE against herpes zoster was lower in older participants, being 64% in participants aged 60–69 years, 41% in participants aged 70–79 years, and 18% in participants aged ≥80 years.^11^ The Long Term Persistence Substudy, which followed a cohort of vaccine recipients from the Shingles Prevention Study for up to 11 years post-vaccination, showed declining efficacy but statistically significant VE against herpes zoster persisting through year 8 post-vaccination.^12^ Subsequent cohort studies have also shown declining effectiveness against the incidence of herpes zoster^13^ but a slower decline against PHN^14^ at 8 years.

In Australia, ZVL is currently indicated for (1) prevention of herpes zoster in individuals 50 years of age and older, and (2) prevention of PHN and reduction of acute and chronic zoster-associated pain in individuals 60 years of age and older.^15^ The Australian National Shingles Vaccination Program, which started on November 1, 2016, provides free herpes zoster vaccination for eligible adults aged 70 years, with a 5-year catch-up program until October 31, 2021 for adults aged 71–79 years, under the National Immunization Program (NIP).^16^ The 70–79 years age group was chosen as the age group likely to receive the greatest benefit from protection against herpes zoster, taking into account the age-specific incidence of herpes zoster and PHN, the decreasing VE with age, the duration of protection provided by the vaccine, and overall cost-effectiveness.^16^ The vaccine is also available on the private market for individuals outside the funded age group and aged over 50 years.

The aim of this report was to evaluate the patterns of ZVL vaccination and the impact of the National Shingles Vaccination Program in Australia since its launch on November 1, 2016. The data shown here offers an interesting comparison to ZVL uptake in the United Kingdom and United States of America.

## Materials and methods

### ZVL vaccination patterns

The maximum uptake of ZVL in adults aged 70–79 years for the period from October 2016 to December 2018 was estimated from (1) the number of ZVL doses provided by Seqirus (Australia) Pty Ltd (distributor for Merck, Sharp & Dohme (Australia) Pty Ltd) to the Australian Department of Health for distribution to general practitioner (GP) practices, and (2) the eligible population based on Australian Bureau of Statistics data for this age group.^17^

Vaccination patterns in adults aged 70–79 years who received ZVL between April 2015 and September 2019 were analyzed using SmartVax® data. The SmartVax system (developed by Ian Peters of DataVation and Dr Alan Leeb) uses short message service (SMS) and smartphone technology to monitor vaccine safety using immunization data extracted from general practice software.^18^ The SmartVax system collects data on patients receiving any vaccine from a subset of GP practices in Australia. At the end of 2018, the active population (≥3 visits in the past 2 years) from the GP practices participating in the SmartVax system constituted 5.5% of the Australian population, ranging from 2.3% in Victoria to 16.3% in Western Australia (2.3–7.2% in the three largest states by population [Victoria, NSW, and Queensland]; Supplementary Table 1).

### Specific antiviral prescriptions for herpes zoster

In Australia, the Pharmaceutical Benefits Scheme (PBS) provides medicines to all Australian residents at a government-subsidized price. The PBS Schedule lists three antiviral drugs to be used exclusively for the treatment of acute herpes zoster in immunocompetent patients: (1) aciclovir (PBS Item No. 01052 J); (2) famciclovir 250 mg (08002E); and (3) valaciclovir (08064 K). These three drugs are restricted to use within 72 hours of the onset of zoster rash and therefore are representative of incident herpes zoster cases. The PBS Schedule also lists famciclovir 500 mg (8897 G); however, it is restricted to use in immunocompromised patients. As ZVL is contraindicated in this population, famciclovir 500 mg was excluded from the analyses.

Data on specific antiviral prescriptions for herpes zoster were analyzed as a surrogate measure to obtain an early indication of the impact of the vaccination program on the incidence of herpes zoster in the target population (70–79 years age group).

Monthly age-specific data on antiviral prescriptions for herpes zoster supplied to a 10% sample of the PBS claims database between January 1, 2006 and December 31, 2018 were provided by the Department of Human Services and extracted and analyzed by Dr Ben Waterhouse (Model Solutions). Only naïve initiations (i.e., where the patient had not previously received a prescription for the drug) were included in the analyses to avoid use in patients with recurrent herpes zoster or use outside the PBS restriction that specifies the treatment is to be administered within 72 hours of the onset of rash.

PBS antiviral prescription data for herpes zoster were analyzed for the following age groups: (1) 60–69 years; (2) 70–79 years; and (3) ≥80 years. Moving annual totals, which are the total number of prescriptions over the course of the previous 12 months, were calculated by adding the number of prescriptions from each new month to the previous 12 months’ total and subtracting the number of prescriptions from the first month of the previous annual period. Prescription rates were calculated by dividing by the Australian population in each age group. Monthly population data were interpolated from annual Australian Bureau of Statistics estimated resident population data.^17^ Prescription rates are presented per million population.

Age-specific trends in specific antiviral prescription rates over time before introduction of the National Shingles Vaccination Program were explored in the three age groups using log-linear regression of the rate of antiviral prescriptions per quarter, with the quarter number as the independent variable and assuming normally distributed residuals on the log scale. The log-linear regression and 95% prediction interval were calculated for each age group using the quarters before the start of the National Shingles Vaccination Program, i.e., before and including Quarter 4 (Q4) 2016, and then extrapolated to the eight subsequent quarters (Q1 2017 to Q4 2018) for which there were data available. The extrapolated prescription rates were assumed to estimate the expected antiviral prescription rates in the absence of vaccination. Once the log-linear regression, calculation of the 95% prediction interval, and extrapolation to the eight subsequent quarters were complete on the log scale, the results were transformed back to the prescription rate scale per million population. Log-linear regression assuming normally distributed errors and corresponding 95% prediction interval were used because the variability of the quarterly frequency data exceeded that estimated by the Poisson distribution.

To provide a quantitative assessment of the impact of the National Shingles Vaccination Program, a segmented log-linear regression was performed to explore age-specific trends in antiviral prescription rates in the three age groups after program implementation. The second segment comprised data from the first quarter in 2017 until the fourth quarter in 2018.

## Results

### ZVL vaccination patterns

From October 2016 to December 2018, 1.487 million doses of ZVL were provided to the Department of Health for distribution to GP practices. The population for herpes zoster vaccination (adults aged 70–79 years) during this period was 1.970 million. On that basis, the maximum possible vaccine uptake in the eligible population was 75%.

During the same period, 125,000 doses were distributed on the private market. If it is assumed that most of these doses were provided to adults aged 60–69 years, a population of 2.450 million in Australia, this would represent an uptake of 5.1%.

**Figure 1.**
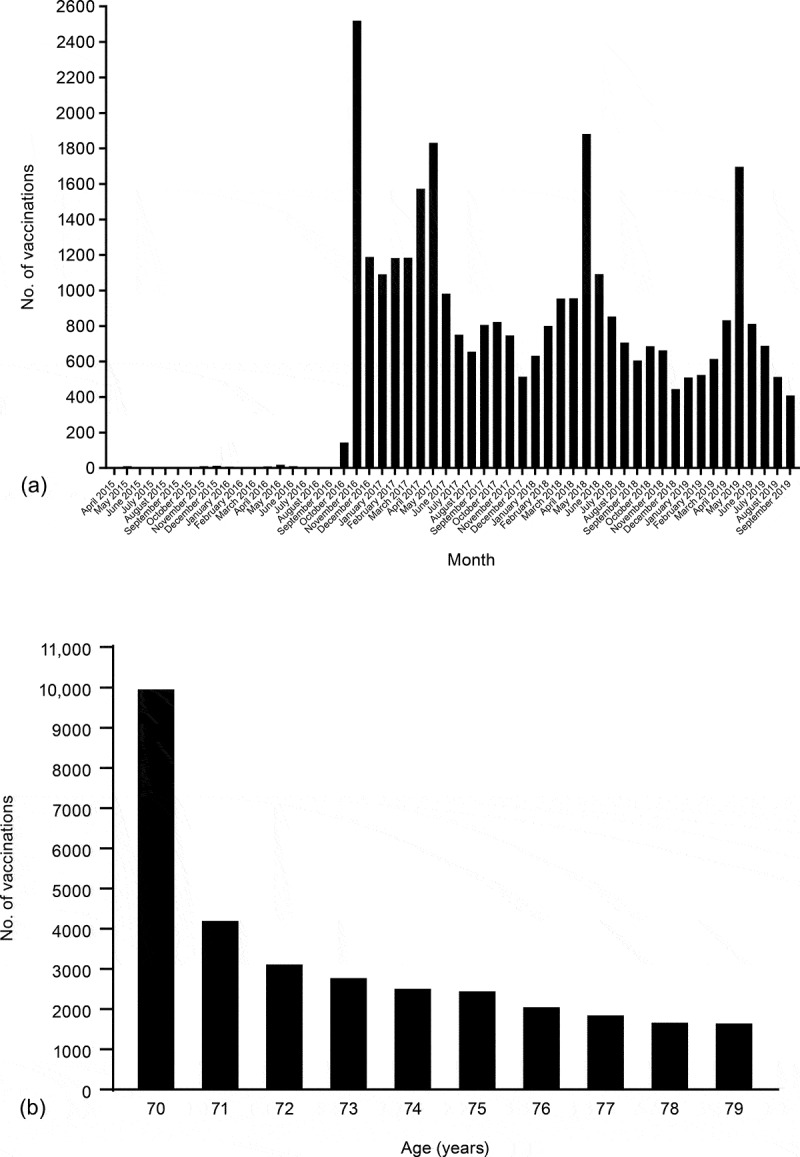
SmartVax ZVL vaccination data. (a) Monthly number of ZVL vaccinations from April 2015 to September 2019. Before commencement of the National Shingles Vaccination Program, between April 2015 and September 2016, between one and 18 doses were recorded per month, which are not discernible in the figure. (b) Number of ZVL vaccinations by age from April 2015 to September 2019

The SmartVax system recorded a total of 33,004 ZVL vaccinations across all indicated age groups between April 2015 and September 2019. Between April 2015 and September 2016, before commencement of the National Shingles Vaccination Program, there were between one and 18 ZVL vaccinations recorded per month (Figure 1(a)), which was indicative of very low use of the vaccine before commencement of the funded program. From November 2016, apart from two months, the number of ZVL vaccinations in the SmartVax population increased to more than 500 per month, with four peaks observed in November 2016, May 2017, May 2018, and May 2019 (Figure 1(a)). The peak in November 2016, with 2520 vaccinations recorded, corresponded with the launch of the National Shingles Vaccination Program on November 1, 2016. The three peaks in May 2017, May 2018, and May 2019, with 1,832, 1,882, and 1,697 vaccinations recorded, respectively, coincided with peaks of influenza vaccination (ZVL can be administered concurrently with inactivated influenza vaccine^15^). While the most common age for ZVL vaccination in the SmartVax system was 70 years (10,030 adults), the majority of patients in the SmartVax system received ZVL vaccination between the ages of 71 and 79 years (22,974 of 33,004 adults, 70%) as part of the catch-up program (Figure 1(b)).

### Analysis of antiviral prescriptions for herpes zoster

**Figure 2.**
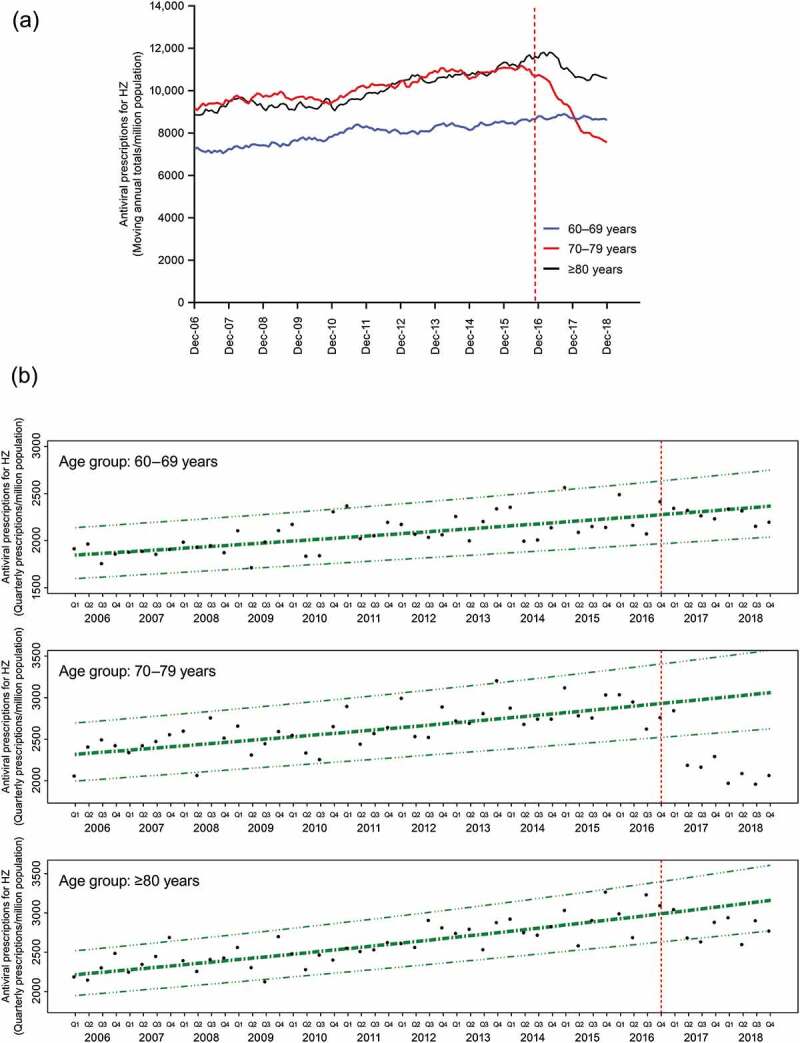
Rates of PBS antiviral prescriptions for herpes zoster over time in the 60–69 years, 70–79 years, and ≥80 years age groups (per million population). (a) Age-specific rates of PBS antiviral prescriptions for herpes zoster are presented as moving annual totals per million population. (b) Age-specific trends in rates of PBS antiviral prescriptions for herpes zoster over time before the introduction of the National Shingles Vaccination Program were analyzed using log-linear regression. Estimated rates per million population are presented in bold dash-dot lines (**− ∙ −**), with 95% prediction intervals in dashed-triple dot lines (− ∙∙∙ −). Observed quarterly rates of PBS antiviral prescriptions for herpes zoster per million population are presented as individual data points (●). Vertical dashed line indicates the start of the National Shingles Vaccination Program

Between 2006 and 2016, there were overall increases in the rate of PBS antiviral prescriptions specific for herpes zoster in each of the three age groups (Figure 2(a)). Before the launch of the National Shingles Vaccination Program on November 1, 2016, the rates of antiviral prescriptions for herpes zoster in the 70–79 years and ≥80 years age groups were similar and higher than in the 60–69 years age group. Following the launch of the vaccination program, there was a marked decrease in the rates of antiviral prescriptions for herpes zoster in the 70–79 years age group, from 10,741 prescriptions per million population in the 12 months to December 2016 to 7,565 prescriptions per million population in the 12 months to December 2018. Rates of antiviral prescriptions for herpes zoster in the 60–69 years age group remained relatively stable over this period (Figure 2(a)). In the ≥80 years age group, which includes participants aged 80–82 years who would have been eligible for vaccination at the start of the National Shingles Vaccination Program, rates of antiviral prescriptions for herpes zoster decreased to a lesser extent than that observed in the 70–79 years age group.

Log-linear regression analysis estimated that between 2006 and 2016, the rate of antiviral prescriptions for herpes zoster increased by 2.0% (95% confidence interval [CI]: 1.3, 2.6), 2.2% (95% CI: 1.5, 2.9) and 2.8% (95% CI: 2.2, 3.4) per year in the 60–69 years, 70–79 years, and ≥80 years age groups, respectively (Table 1). Following implementation of the National Shingles Vaccination Program, in the eligible population aged 70–79 years, the antiviral prescription rates observed in the last seven quarters (corresponding to the period April 2017 to December 2018) fell below the lower limit of the 95% prediction interval and well below the extrapolated line of best fit from the log-linear regression (Figure 2(b)). This finding indicates that there was a substantial decrease in the rates of antiviral prescriptions for herpes zoster compared with predictions based on trends observed before implementation of the vaccination program. In contrast, in the 60–69 years age group, not eligible for the funded program, all observed antiviral prescription rates fell within the 95% prediction intervals (Figure 2(b)). In the ≥80 years age group, which includes participants aged 80–82 years who would have been eligible for vaccination at the start of the National Shingles Vaccination Program, the antiviral prescription rates observed in four of the last eight quarters fell on or below the lower limit of the prediction interval (Figure 2(b)). Additional analysis was conducted in the ≥82 years age group, that is, excluding participants aged 80 or 81 years old who would have been eligible for vaccination at the start of the program. In the ≥82 years age group, all observed antiviral prescription rates fell within the 95% prediction intervals (Supplementary Figure 1).

**Table 1.** Estimated trends in age-specific antiviral prescriptions for herpes zoster

	Pre- Shingles Vaccination Program (Q1 2006 – Q4 2016)		Post- Shingles Vaccination Program (Q1 2017 – Q4 2018)	
Age group	Prescription Rate Ratio (95% CI)	Change per year (95% CI)	Prescription Rate Ratio (95% CI)	Change per year (95% CI)
60 – 69 years	1.020	2.0%	0.966	−3.4%
	(1.013, 1.026)	(1.3%, 2.6%)	(0.929, 1.004)	(−7.1%, 0.4%)
70–79 years	1.022	2.2%	0.864	−13.6%
	(1.015, 1.029)	(1.5%, 2.9%)	(0.758, 0.984)	(−24.2%, −1.5%)
80+ years	1.028	2.8%	0.987	−1.3%
	(1.022, 1.034)	(2.2%, 3.4%)	(0.899, 1.083)	(−10.1%, 8.3%)

The above findings were confirmed by segmented log-linear regression, which estimated that in the two years since program launch, herpes zoster antiviral prescription rates declined significantly by an average of 13.6% per year (95% CI: 1.5, 24.2) in the eligible population aged 70–79 years. In contrast, no significant trends were observed for the other age groups (Table 1).

## Discussion

The burden of disease from herpes zoster is considerable and rising as the population ages. Treatment of acute herpes zoster with antiviral drugs ameliorates the severity and duration of the disease, but a considerable impact remains on the patient’s quality of life. Vaccine prevention of herpes zoster is therefore the best strategy to help reduce the burden of the disease. ZVL is the first vaccine for herpes zoster and the first new vaccine for older people listed on the Australian NIP for over 20 years. We report here the first evaluation of the impact of the National Shingles Vaccination Program on the incidence of herpes zoster in Australia. The current analyses suggest good uptake of the vaccine in adults aged 70–79 years since the launch of the program on November 1, 2016; this is associated with a marked decrease in antiviral prescriptions for herpes zoster (a surrogate for the incidence of herpes zoster) in the 70–79 years age group that was not observed in the 60–69 years age group and was observed to a lesser extent in the ≥80 years age group, which includes participants aged 80–82 years who would have been eligible for vaccination at the start of the National Shingles Vaccination Program. These data support the effectiveness of ZVL to reduce the incidence of herpes zoster in the target population.

On the basis of the number of doses of ZVL distributed during the first 2 years of the Australian National Shingles Vaccination Program, the maximum possible uptake in the target population was estimated to be 75%. However, we note that this calculation does not account for doses held in stock, expired doses, and/or wastage, and thus is likely an overestimate of the true vaccine coverage. Using the Australian Immunization Register (AIR), the National Center for Immunization Research and Surveillance (NCIRS) estimated coverage in Australia of 33.9% in 70-year-old adults and 25.8% in adults aged 71–79 years from November 1, 2016 to March 31, 2018.^16^ During this period, 1,370,395 doses of ZVL were distributed under the NIP, but only 489,605 doses were reported in the AIR, indicating substantive underreporting of the administration of the vaccine. In addition, data from the Department of Health (Australia) suggest that vaccine wastage, due in part to cold chain deviations, is approximately 10%.^19^ Given these assumptions, the actual coverage of ZVL in Australia is therefore likely to be estimated at 60–65%, below the maximum uptake of 75% reported here, but well above the coverage of 33.9%/25.8% reported by NCIRS. This estimate is in line with that reported in the United Kingdom, which also has a publicly funded herpes zoster vaccination program, and where herpes zoster vaccine coverage was reported to be 63%, 61%, and 58% in three routine cohorts (adults aged 70 years) and 60%, 58%, 59%, and 61% in four catch-up cohorts (adults aged 78 or 79 years) during the first 3 years of a herpes zoster vaccination program (2013–2016).^20^ In contrast, in the United States, which does not have a publicly funded herpes zoster vaccination program, relatively low but rising levels of live herpes zoster vaccine coverage have been reported in adults aged ≥60 years, increasing from 1.3% in 2007 to 19.5% in 2013^21^ and reaching 33.4% in 2016.^22^ Factors such as the cost of the vaccine and complex methods for reimbursement have been reported as financial barriers to the uptake of the herpes zoster vaccine in the United States.^2,23-25^ The private market data reported here show that although the herpes zoster vaccine is available on the private market for individuals outside the funded age group in Australia, it is not taken up in great numbers (5%). Similarly, SmartVax data show very low uptake of the vaccine in the 70–79 years age group on the private market before implementation of the National Shingles Vaccination Program. In addition, while early shortages of the vaccine that affected uptake in Australia^16^ and the United States^26^ have been resolved, other barriers to the uptake of herpes zoster vaccine have been reported, including low perceived risk of getting shingles, concern about the effectiveness and/or safety of the vaccine, and belief that the herpes zoster vaccine can cause shingles (Table 2).^27^ The progressive decline in uptake over the 5 years of the herpes zoster vaccination program in the United Kingdom^20,28^ highlights the need to implement strategies to overcome barriers to uptake of the herpes zoster vaccine.

**Table 2.** Factors influencing the uptake of the herpes zoster vaccine.^a^

Factors associated with decreased uptake of herpes zoster vaccine	
Beliefs about shingles or immunity	Low perceived risk of getting herpes zoster
	Belief that vaccine is not needed/rarely get sick
	Belief that they already have good immunity to herpes zoster
	Belief that natural immunity is better/vaccines weaken the immune system
Beliefs about the herpes zoster vaccine	Concerns about the effectiveness of the herpes zoster vaccine
	Concerns about adverse effects from the herpes zoster vaccine
	Concerns about a possible allergic reaction to the herpes zoster vaccine
	Belief that the herpes zoster vaccine can cause shingles
Healthcare provider	GP has not discussed the need for the herpes zoster vaccine
	Difficulty getting to see GP
Factors associated with increased uptake of herpes zoster vaccine	
Demographic	Older age
	Female
	Higher level of education
Health knowledge and behavior	Regularly gets influenza or pneumococcal vaccines
	Higher awareness of shingles and the herpes zoster vaccine
Beliefs about herpes zoster	Belief that herpes zoster can be a severe condition
Healthcare provider	Has a usual GP
	Strong recommendation from GP to get the herpes zoster vaccine
Other	Family or friends have previously been affected with herpes zoster or PHN
	Herpes zoster vaccine available

GP, general practitioner; PHN, postherpetic neuralgia.

^a^Adapted from Litt et al.^27^

Factors associated with decreased/increased uptake of the herpes zoster vaccine are shown in Table 2. Strategies targeting these factors that have had an impact on the uptake of the herpes zoster vaccine include GP education to increase awareness that shingles is a vaccine-preventable disease and that influenza vaccination is an optimal time to consider shingles vaccination. Analysis of the SmartVax monthly vaccination data from April 2015 to September 2019 showed that the highest number of vaccinations occurred in November 2016, the month the National Shingles Vaccination Program was launched. The launch of the program was preceded by an extensive direct mail-out of communication materials to vaccine providers to raise awareness of the program and provide education on the vaccine, including how to order and store the vaccine.^16^ There was also a series of activities during the launch, including: extensive national peer-to-peer education; scientific and expert advisory board meetings; third-party and Continuing Professional Development GP educational programs; and healthcare provider recall programs to help identify eligible adults aged 70 years and older who may be considered for the ZVL vaccine. The second to fourth highest number of vaccinations were reported in May 2017, May 2018, and May 2019, which suggests that herpes zoster vaccination was linked to the annual influenza vaccination in Australia during 2017, 2018, and 2019. A randomized controlled trial has shown that the herpes zoster vaccine may be administered concurrently with the inactivated influenza vaccine, with similar immune responses and safety profiles observed in the group who received the two vaccines simultaneously and the group who received the two vaccines individually, separated by 4 weeks.^11,15,29^ In addition, further primary care awareness campaigns on herpes zoster and ZVL were conducted and likely contributed to the three peaks in herpes zoster vaccination observed in May 2017, May 2018, and May 2019. Other strategies that may improve uptake of the herpes zoster vaccine include addressing the concerns and misperceptions of patients and GPs, identifying and directly targeting the eligible population by flagging electronic records of eligible patients to indicate when the vaccine is due and when it has been received, systematic enquiry by GPs and practice nurses, targeting infrequent attenders (who are less likely to receive preventive activities), and health reminder services (e.g., SmartVax, HotDoc). As GP recommendation has been identified as one of the strongest predictors of patient intention to get the herpes zoster vaccine,^30^ strategies to enhance GP recommendation of the vaccine are likely to have the greatest influence on its uptake.

There was a marked decrease in antiviral prescriptions specific for herpes zoster in the 70–79 years age group following the launch of the National Shingles Vaccination Program in November 2016, indicating a decrease in the incidence of herpes zoster in this age group. In the absence of the vaccination program, it is likely that the increasing trend in antiviral prescriptions in the 70–79 years age group would have continued, as it has in the 60–69 years age group. A decrease in antiviral prescriptions for herpes zoster following the launch of the vaccination program was also observed for some time-points in the ≥80 years age group, but the trend was not statistically significant. This decrease is likely to reflect, at least in part, the fact that participants aged 80–82 years would have been eligible for vaccination at the start of the program; a proportion of these participants would likely have received the herpes zoster vaccine before turning 80 years old.

The observed marked decrease in antiviral prescriptions for herpes zoster in the eligible population aged 70–79 in the two years since the introduction of the National Shingles Vaccination Program was confirmed by segmented log-linear regression, which estimated a statistically significant 13.6% (1.5, 24.2) average decrease per year in the rate of antiviral prescriptions in this age group. This result must be interpreted with caution, as it represents a relatively short follow-up period after the introduction of the vaccine, and ongoing studies are required to determine whether this trend will be maintained.

Although the effect of the vaccination program on the incidence of herpes zoster in the 70–79 years age group could not be directly assessed in these analyses, the observed decrease in antiviral prescriptions for herpes zoster in the population eligible for vaccination supports the effectiveness of the vaccine. Our results are consistent with findings in the United Kingdom, where a decrease in the incidence of herpes zoster was observed during the first 3 years of the herpes zoster vaccination program using linked individual-level electronic health records.^31^ The incidence of herpes zoster in the analyses from the United Kingdom was 3.15/1,000 person years in vaccinated individuals compared with 8.80/1,000 person years in unvaccinated individuals, resulting in an estimate of vaccine effectiveness against herpes zoster of 64%.^31^

Before the launch of the National Shingles Vaccination Program, we found a trend for increasing numbers of antiviral prescriptions for herpes zoster in each of the 60–69 years, 70–79 years, and ≥80 years age groups, indicating an increase in the incidence of herpes zoster between 2006 and 2016. An increase over time in the incidence of herpes zoster in Australia has been reported previously, as assessed by PBS data on antiviral prescriptions for herpes zoster and general practice encounters with herpes zoster.^3,32^ Increased incidence of herpes zoster over time has also been reported in other parts of the world.^33^ Possible reasons for the recent increasing incidence of herpes zoster in the absence of vaccination include the aging of the population and the increased use of immunosuppressant drugs.^2,33^ In addition, it has been suggested that the introduction of routine varicella (chickenpox) vaccination in children (in 2005 in Australia) may reduce the re-exposure to VZV needed to boost declining cell-mediated immunity in adults.^2,3,33,34^ However, data on this are conflicting, as at least some of the increase in herpes zoster incidence preceded the introduction of the varicella vaccination program.^2,3,33,34^

This study assumes that the trends in specific antiviral prescriptions for herpes zoster reflect trends in herpes zoster incidence, noting that the specific antiviral drugs are restricted to use within 72 hours of the onset of rash, indicating incident herpes zoster cases. This approach has been previously validated by comparison with BEACH analyses.^3^ However, not all patients with herpes zoster receive timely antiviral drug treatment, with previous analyses of the BEACH database reporting antiviral prescriptions in only 69% of GP encounters for new herpes zoster problems in patients aged 50 years and older.^3^ The numbers of antiviral prescriptions for herpes zoster thus underestimate the total number of herpes zoster cases in Australia. Collection of BEACH data ceased in 2017, precluding updated analysis of general practice antiviral prescription patterns. This is a limitation of this study, which does not attempt to estimate age-specific herpes zoster incidence. The underlying assumption is that antiviral prescription patterns have remained relatively constant over time, and thus the trends in antiviral prescriptions for herpes zoster are representative of trends in the incidence of herpes zoster.

As discussed, a limitation of the uptake estimate is that the analyses do not take into account the doses held in stock by the government, expired doses, and/or wastage, and thus it is likely to be an overestimate. In addition, although the SmartVax data provide an indicator of vaccine uptake in a sample of Australian clinics, there was no available denominator to allow calculation of ZVL uptake in the SmartVax population. The SmartVax system has subsequently been updated to permit reporting of the denominator, and thus more comprehensive analyses of ZVL uptake may be conducted in the future. Although the SmartVax population covered a reasonable sample of the Australian population (5.5%), it was not evenly distributed across the states/territories and therefore may not be representative of the eligible population in Australia. In the absence of data linkage analyses to formally assess vaccine effectiveness, as reported for the United Kingdom herpes zoster vaccination program,^31^ the analyses reported here still allow assessment of the initial impact of the National Shingles Vaccination Program in Australia.

Herpes zoster is of major importance to the aging Australian population because of its morbidity (in particular, PHN), which increases in incidence with age. The current analyses show that, during its first 2 years, the National Shingles Vaccination Program has been highly successful in vaccinating a considerable proportion of eligible Australian adults aged 70–79 years against herpes zoster. The marked decrease in antiviral prescriptions for herpes treatment in the 70–79 years age group following the launch of the vaccination program suggests that uptake of the vaccine was associated with a decrease in the incidence of herpes zoster in this population.

## Supplementary Material

Supplemental Material

## References

[1] Cunningham AL, Breuer J, Dwyer DE, Gronow DW, Helme RD, Litt JC, Levin MJ, Macintyre CR. The prevention and management of herpes zoster. Med J Aust. 2008;188:171–76. doi:10.5694/j.1326-5377.2008.tb01566.x. 18241179

[2] Forbes HJ, Thomas SL, Langan SM. The epidemiology and prevention of herpes zoster. Curr Dermatol Rep. 2012;1:39–47. doi:10.1007/s13671-011-0004-4.

[3] MacIntyre R, Stein A, Harrison C, Britt H, Mahimbo A, Cunningham A. Increasing trends of herpes zoster in Australia. PLoS One. 2015;10:e0125025. doi:10.1371/journal.pone.0125025. 25928713 PMC4416021

[4] Johnson RW, Dworkin RH. Treatment of herpes zoster and postherpetic neuralgia. BMJ. 2003;326:748–50. doi:10.1136/bmj.326.7392.748. 12676845 PMC1125653

[5] Langan SM, Minassian C, Smeeth L, Thomas SL. Risk of stroke following herpes zoster: a self-controlled case-series study. Clin Infect Dis. 2014;58:1497–503. doi:10.1093/cid/ciu098. 24700656 PMC4017889

[6] Minassian C, Thomas SL, Smeeth L, Douglas I, Brauer R, Langan SM. Acute cardiovascular events after herpes zoster: a self-controlled case series analysis in vaccinated and unvaccinated older residents of the United States. PLoS Med. 2015;12:e1001919. doi:10.1371/journal.pmed.1001919. 26671338 PMC4682931

[7] Zhang Y, Luo G, Huang Y, Yu Q, Wang L, Li K. Risk of stroke/transient ischemic attack or myocardial infarction with herpes zoster: a systematic review and meta-analysis. J Stroke Cerebrovasc Dis. 2017;26:1807–16. doi:10.1016/j.jstrokecerebrovasdis.2017.04.013. 28501259

[8] Erskine N, Tran H, Levin L, Ulbricht C, Fingeroth J, Kiefe C, Goldberg RJ, Singh S. A systematic review and meta-analysis on herpes zoster and the risk of cardiac and cerebrovascular events. PLoS One. 2017;12:e0181565. doi:10.1371/journal.pone.0181565. 28749981 PMC5531458

[9] Stein AN, Britt H, Harrison C, Conway EL, Cunningham A, Macintyre CR. Herpes zoster burden of illness and health care resource utilisation in the Australian population aged 50 years and older. Vaccine. 2009;27:520–29. doi:10.1016/j.vaccine.2008.11.012. 19027048

[10] Oxman MN, Levin MJ, Johnson GR, Schmader KE, Straus SE, Gelb LD, Arbeit RD, Simberkoff MS, Gershon AA, Davis LE, et al. A vaccine to prevent herpes zoster and postherpetic neuralgia in older adults. N Engl J Med. 2005;352:2271–84. doi:10.1056/NEJMoa051016. 15930418

[11] ZOSTAVAX® (Zoster Vaccine Live) [prescribing information]. Whitehouse Station (NJ): Merck & Co., Inc.; 2019 [accessed 2019 Jun 12]. https://www.merck.com/product/usa/pi_circulars/z/zostavax/zostavax_pi2.pdf.

[12] Morrison VA, Johnson GR, Schmader KE, Levin MJ, Zhang JH, Looney DJ, Betts R, Gelb L, Guatelli JC, Harbecke R, et al. Long-term persistence of zoster vaccine efficacy. Clin Infect Dis. 2015;60:900–09. doi:10.1093/cid/ciu918. 25416754 PMC4357816

[13] Tseng HF, Harpaz R, Luo Y, Hales CM, Sy LS, Tartof SY, Bialek S, Hechter RC, Jacobsen SJ. Declining effectiveness of herpes zoster vaccine in adults aged ≥60 years. J Infect Dis. 2016;213:1872–75. doi:10.1093/infdis/jiw047. 26908728

[14] Klein NP, Bartlett J, Fireman B, Marks MA, Hansen J, Lewis E, Aukes L, Saddier P. Long-term effectiveness of zoster vaccine live for postherpetic neuralgia prevention. Vaccine. 2019;37:5422–27. doi:10.1016/j.vaccine.2019.07.004. 31301920

[15] ZOSTAVAX® (Zoster Virus Vaccine Live [Oka/Merck] Refrigerator stable) [Australian product information]. Macquarie Park (NSW): Merck Sharp & Dohme (Australia) Pty Limited; 2019 [accessed 2019 Jun 12]. https://www.ebs.tga.gov.au/ebs/picmi/picmirepository.nsf/pdf?OpenAgent&id=CP-2010-PI-01547-3&d=201906121016933.

[16] National Centre for Immunisation Research and Surveillance (NCIRS). Evaluation of the National Shingles vaccination program process and early impact evaluation. Final Report. 2019 Mar 1 [accessed 2019 Jun 7]. http://ncirs.org.au/sites/default/files/2019-04/Shingles%20Program%20Evaluation%20Report_1%20March%202019_Final%20for%20web.pdf.

[17] Australian Bureau of Statistics. 3101.0 – Australian demographic statistics. TABLE 59. Estimated Resident Population By Single Year Of Age, Australia; 2018 Dec [accessed 2019 Aug 1]. https://www.abs.gov.au/AUSSTATS/abs@.nsf/DetailsPage/3101.0Dec%202018?OpenDocument.

[18] Leeb A, Regan AK, Peters IJ, Leeb C, Leeb G, Effler PV. Using automated text messages to monitor adverse events following immunisation in general practice. Med J Aust. 2014;200:416–18. doi:10.5694/mja13.11166. 24794676

[19] Department of Health, Commonwealth of Australia. National immunisation strategy for Australia 2019–2024. 2019 Feb 17 [accessed 2019 Sep 20]. https://www.health.gov.au/resources/publications/national-immunisation-strategy-for-australia-2019-to-2024.

[20] Amirthalingam G, Andrews N, Keel P, Mullett D, Correa A, de Lusignan S, Ramsay M. Evaluation of the effect of the herpes zoster vaccination programme 3 years after its introduction in England: a population-based study. Lancet Public Health. 2018;3:e82–e90. doi:10.1016/S2468-2667(17)30234-7. 29276017 PMC5846879

[21] Zhang D, Johnson K, Newransky C, Acosta CJ. Herpes zoster vaccine coverage in older adults in the U.S., 2007–2013. Am J Prev Med. 2017;52:e17–e23. doi:10.1016/j.amepre.2016.08.029. 28340974

[22] Centers for Disease Control and Prevention. Vaccination coverage among adults in the United States, National health interview survey. 2016 [accessed 2019 Jun 13]. https://www.cdc.gov/vaccines/imz-managers/coverage/adultvaxview/pubs-resources/NHIS-2016.html.

[23] Hurley LP, Harpaz R, Daley MF, Crane LA, Beaty BL, Barrow J, Babbel C, Marin M, Steiner JF, Davidson A, et al. National survey of primary care physicians regarding herpes zoster and the herpes zoster vaccine. J Infect Dis. 2008;197(Suppl 2):S216–223. doi:10.1086/522153. 18419400

[24] Hurley LP, Lindley MC, Harpaz R, Stokley S, Daley MF, Crane LA, Dong F, Beaty BL, Tan L, Babbel C, et al. Barriers to the use of herpes zoster vaccine. Ann Intern Med. 2010;152:555–60. doi:10.7326/0003-4819-152-9-201005040-00005. 20439573

[25] High KP. Overcoming barriers to adult immunization. J Am Osteopath Assoc. 2009;109:S25–28. 19553634

[26] Williams WW, Lu PJ, O’Halloran A, Kim DK, Grohskopf LA, Pilishvili T, Skoff TH, Nelson NP, Harpaz R, Markowitz LE, et al. Surveillance of vaccination coverage among adult populations – united States, 2015. MMWR Surveill Summ. 2017;66:1–28. doi:10.15585/mmwr.ss6611a1. PMC5829683 28472027

[27] Litt J, Cunningham T, Van Buynder P Update on herpes zoster. Healthed expert monograph, issue 18. 2018 [accessed 2019 Oct 2]. https://www.healthed.com.au/monographs/update-herpes-zoster/.

[28] Public Health England. Herpes zoster (shingles) immunisation programme 2017 to 2018: evaluation report. Evaluation of the fifth year of the shingles vaccination programme in England from 2017 to 2018. Health Protection Report. Volume 12 Number 42; 2018.

[29] Kerzner B, Murray AV, Cheng E, Ifle R, Harvey PR, Tomlinson M, Barben JL, Rarrick K, Stek JE, Chung MO, et al. Safety and immunogenicity profile of the concomitant administration of ZOSTAVAX and inactivated influenza vaccine in adults aged 50 and older. J Am Geriatr Soc. 2007;55:1499–507. doi:10.1111/j.1532-5415.2007.01397.x. 17908055

[30] Litt JCB, Kim S, Woodman R, MacIntyre R, Cunningham T. Australian zoster study: GP and patient views about herpes zoster (shingles), its complications, and the likely acceptance of a zoster vaccine (Zostavax). Int J Infect Dis. 2014;21(Suppl 1):436–37. doi:10.1016/j.ijid.2014.03.1320.

[31] Walker JL, Andrews NJ, Amirthalingam G, Forbes H, Langan SM, Thomas SL. Effectiveness of herpes zoster vaccination in an older United Kingdom population. Vaccine. 2018;36:2371–77. doi:10.1016/j.vaccine.2018.02.021. 29555217 PMC5899761

[32] Jardine A, Conaty SJ, Vally H. Herpes zoster in Australia: evidence of increase in incidence in adults attributable to varicella immunization? Epidemiol Infect. 2011;139:658–65. doi:10.1017/S0950268810001949. 20727248

[33] Kawai K, Gebremeskel BG, Acosta CJ. Systematic review of incidence and complications of herpes zoster: towards a global perspective. BMJ Open. 2014;4:e004833. doi:10.1136/bmjopen-2014-004833. PMC4067812 24916088

[34] Reynolds MA, Chaves SS, Harpaz R, Lopez AS, Seward JF. The impact of the varicella vaccination program on herpes zoster epidemiology in the United States: a review. J Infect Dis. 2008;197(Suppl 2):S224–227. doi:10.1086/522162. 18419401

