# Supplementary figures and images for: Early impact of the Australian national shingles vaccination program with the herpes zoster live attenuated vaccine
Source: Hum Vaccin Immunother. 2020 May 18;16(12):3081–9. doi: 10.1080/21645515.2020.1754702 (PMC8641585; doi:10.1080/21645515.2020.1754702)

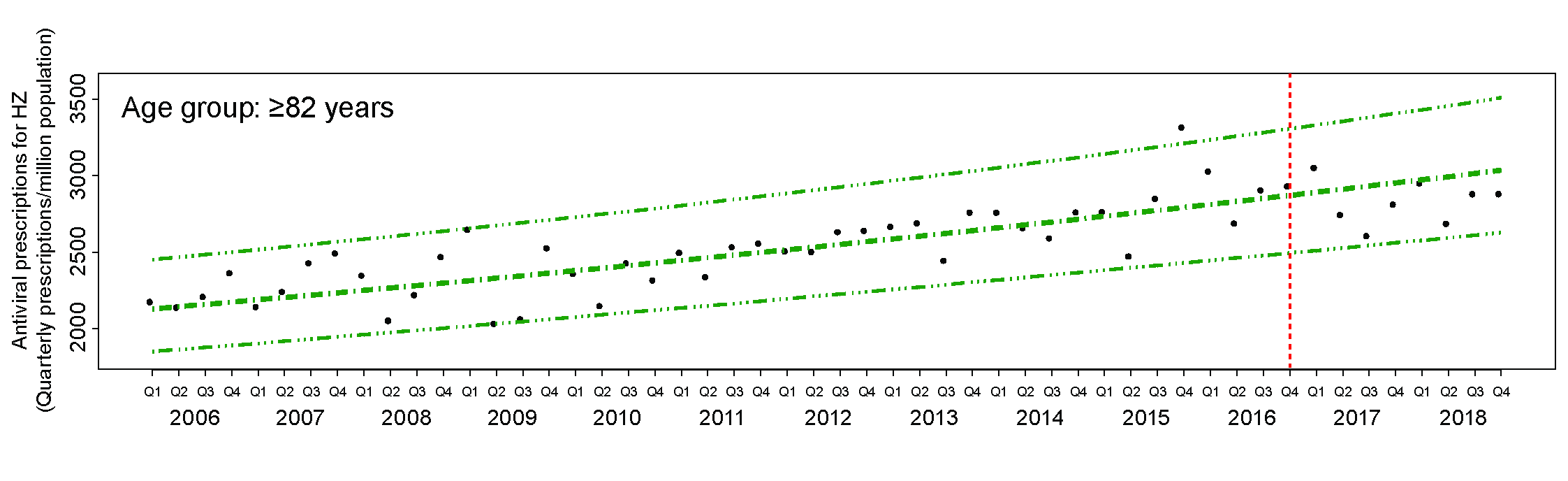

Supplement: Supplemental Material [file KHVI_A_1754702_SM1909.tif]
